# Safety and Efficacy of Zavegepant in Treating Migraine: A Systematic Review

**DOI:** 10.7759/cureus.41991

**Published:** 2023-07-17

**Authors:** Zainab Z Khan, Usman Ahmed, Faizan Shahzad, Muaz Ali, Kashif Tousif, Usman Ahmed, Qazi Muhammad Safwan, Syed Imam Naufil, Sara Murtaza, Sajeel Saeed, Jawad Basit, Tehseen Haider, Haroon Shabbir

**Affiliations:** 1 Neurology, CMH (Combined Military Hospital) Lahore Medical College and Institute of Dentistry, Lahore, PAK; 2 Pediatric Surgery, Rawalpindi Medical University, Rawalpindi, PAK; 3 Cardiology, Rawalpindi Medical University, Rawalpindi, PAK; 4 Internal Medicine, Rawalpindi Medical University, Rawalpindi, PAK; 5 Surgery, Rawalpindi Medical University, Rawalpindi, PAK; 6 Cardiology, Allama Iqbal Medical College, Lahore, PAK; 7 Neurology, Hayatabad Medical Complex, Peshawar, PAK; 8 Neurology, Rawalpindi Medical University, Rawalpindi, PAK

**Keywords:** migraine treatment, migraine management, intranasal migraine drug, gepants, therapeutic agent, trigemino-vascular system, international headache society, calcitonin gene-related peptide (cgrp) pathway, zavegepant, migraine headaches

## Abstract

Drugs that act on the calcitonin gene-related peptide (CGRP) pathway herald the dawn of a new era in the management of migraine headaches. The blockade of CGRP alleviates neural inflammation and has been associated with reduced pain sensitization. Zavegepant is a third-generation drug and is the first intranasal CGRP antagonist to be developed. This systematic review aims to assess the safety, efficacy, pharmacokinetics, and tolerability of Zavegepant as an abortive treatment for migraine. Studies that assessed the safety, tolerability, and efficacy of Zavegepant for migraine were identified through a systematic literature review of PubMed, Clinicaltrials.gov, and Cochrane databases in April 2023. Our systematic review yielded a total of six studies that fit our inclusion criteria. Of these, data from only two randomized control trials (RCTs) was homogenous; hence, forest plots of results pooled from the included studies were not reported. The included studies showed that Zavegepant is an efficacious and well-tolerated abortive treatment modality for episodic migraine in adult patients. Zavegepant showed safety and efficacy in migraine treatment according to various parameters throughout the six included studies. These parameters include adverse events, pharmacokinetic properties, CGRP inhibition, effect on blood pressure/electrocardiogram, pain freedom, and freedom from most bothersome symptoms.

## Introduction and background

Migraine is a primary headache disorder most often characterized by a unilateral headache, with or without aura [1]. It is well established that migraines have a genetic basis and more than 80% of people suffering from migraine headaches have a first-line relative with the same condition [2]. Several factors can trigger a migraine attack, including stress, certain sensory stimuli, alcohol, contraceptives, vasodilators, food additives, e.g., aspartame, and sleep changes. Women are significantly more likely to develop migraines as compared to men, possibly triggered by hormone changes during menstruation [3]. Migraine headaches are one of the most common causes of emergency hospital visits, accounting for 3% of such visits annually in the United States [4]. The major subtypes of migraine headaches recognized by the headache classification committee of the International Headache Society include migraine with or without aura and chronic migraine. Migraine with aura is characterized by unilateral, recurrent attacks, lasting for a few minutes, accompanied by some form of visual, auditory, or other central nervous system symptoms that are fully reversible. Migraine without aura, previously known as “hemicrania simplex”, is characterized by a unilateral headache of moderate to severe intensity with a pulsating nature, associated with some degree of photophobia, phonophobia, and/or nausea [5]. Chronic headache is characterized by a headache occurring on 15 or more days per month, persisting for more than three months. It is defined as chronic when it fits the above-mentioned criteria and has the features of a migraine attack on more than eight days per month [5].

Activation of the trigeminovascular system signals the release of pain-inducing neuropeptides, which cause dilatation of extracranial vessels, leading to the throbbing sensation of a migraine headache [6]. Various vasoactive neuropeptides have been found associated with the pathogenesis of migraine headaches, including substance P, neurokinin a, pituitary adenylate cyclase-activating polypeptide, and calcitonin gene-related peptide (CGRP), with elevated levels of said neuropeptides found in the spinal fluid of people suffering from migraines [7,8]. A systematic review and meta-analysis by Frederiksen et al. to identify relevant biomarkers in migraine and cluster headache patients reported higher interictal CGRP blood levels in episodic and chronic migraines along with higher ictal CGRP blood levels in episodic migraine [9]. CGRP is abundant in the trigeminal system and is known to induce vasodilation and trigeminal sensitization [10]. Hence, a potential target for therapeutic agents is the CGRP receptor. A category of drugs that target this receptor is “Gepants”, of which zavegepant is a promising candidate drug administered intranasally [11].

Therapeutic agents commonly used for the management of migraine headaches include triptans, non-steroidal anti-inflammatory drugs (NSAIDs), antiemetics, acetaminophen, and ergotamine, with triptans (sumatriptan) being the first line of treatment, especially for patients with allodynia [1,12]. CGRP antagonists provide a promising therapeutic action for patients with migraine and have shown efficacy in various clinical trials.

We conducted a systematic review of PubMed, Clinicaltrials.gov, and Cochrane databases to determine the safety and efficacy of zavegepant, which is a relatively new CGRP antagonist and has an advantage of intranasal administration. This promises to be a key development in migraine treatment, especially for people who prefer not to take oral medications [11].

## Review

Material and methods

Search Strategy

**Figure 1:**
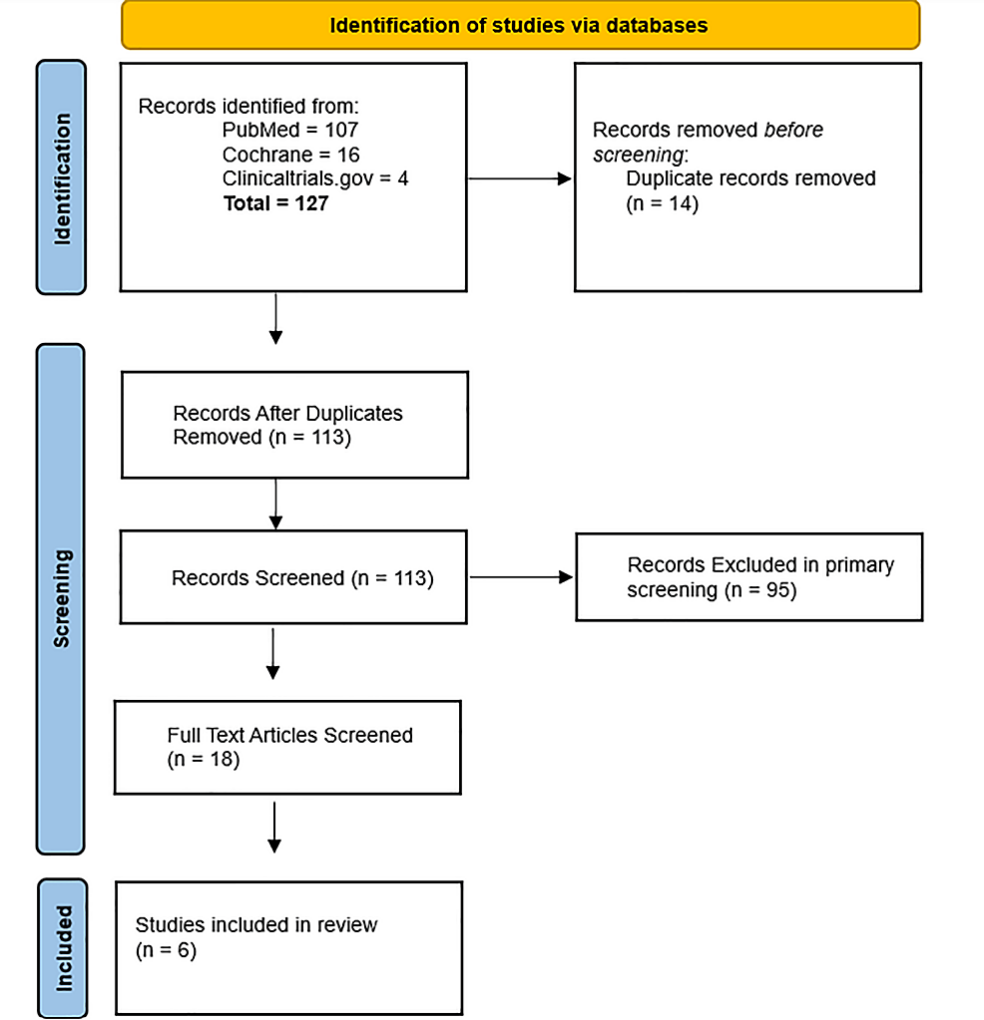
PRISMA Flow Chart PRISMA: Preferred Reporting Items for Systematic Reviews and Meta-Analyses

A systematic literature search was performed in the PubMed, Embase, Cochrane, and Clinicaltrials.gov databases from inception till April 22, 2023. Medical Subject Headings (MeSH) keywords and all relevant words relating to zavegepant and migraine were utilized. To identify articles that were overlooked during the initial screening, we used backward snowballing by screening the reference lists of relevant articles. By doing so, additional articles that were not included in the initial search can be identified, along with inconsistencies in the literature. Moreover, we applied no time filters to the search. Our study was conducted in accordance with the Preferred Reporting Items for Systematic Reviews and Meta-Analyses (PRISMA) guidelines. The PRISMA flow diagram for primary and secondary screening is shown in Figure 1.

Eligibility Criteria

Our search resulted in 127 articles, of which 14 were removed because they were duplicates. After the primary screening, 95 records were removed because they didn’t meet the eligibility criteria. Eighteen articles were screened for full text. Of these, only six were included in the systematic review. We included all clinical trials that evaluated the safety, tolerability, pharmacokinetics, and efficacy of zavegepant for the treatment of migraine. Conference abstracts were also included if they provided sufficient data about the outcomes. All editorials, case reports, case series, and controlled observational studies (cross-sectional, case-control, cohort studies) were excluded. Studies published in languages other than English were also excluded. All studies conducted on animals, studies performed on healthy adults, studies not using the CGRP inhibitor zavegepant, and studies not reporting primary and secondary outcomes of interest were excluded.

Study Selection

Two authors (HS and FS) scrutinized the titles and abstracts of all articles obtained from the literature search and removed irrelevant articles. The full texts of the remaining articles were evaluated by two different authors (SS and UA), and studies that met the predefined inclusion criteria were included in the analysis. In case of any discrepancies, another author (ZZK) was consulted, and disagreements were resolved through mutual discussion.

Data Extraction and Quality Assessment

A data collection form, which included all relevant variables and study outcomes, was created using Microsoft Excel (Microsoft Corporation, Redmond, Washington, United States). Two authors (MA and KT) independently assessed each included article, and extracted data related to the clinical trial phase, year of study, sample size, baseline characteristics, primary outcomes, and any adverse events that were reported. The baseline characteristics of the included studies have been tabulated in Table 1. 

**Table 1:** Baseline characteristics of included studies C_max_: maximum concentration; AUC0-inf: area under curve on concentration-time graph from time 0 to infinity; CGRP: calcitonin gene-related peptide; F: Female; M: Male; NA: not applicable; AUC: area under curve; PK: pharmacokinetics

Author, Year, and Title	Participant flow	Mean Age (SD)/ Range	Sex	Prophylactic Migraine Medication Use at Randomization	Outcome Measures	Main Findings
Bertz et al., 2022 [13] “Safety, Tolerability, and Pharmacokinetics of Single and Multiple Ascending Doses of Intranasal Zavegepant in Healthy Adults”	144 enrolled, 72 each in single and multiple dose studies	18-55	NA	NA	C_max, _ AUC, CGRP inhibition	Single doses of zavegepant greater than 10mg produced a C_max_ associated with greater efficacy. Doses up to 40 mg were found to be safe and well tolerated
Bertz et al., 2022 [14] "Comparative Bioavailability of Single-Dose Zavegepant Nasal Spray During and Between Migraine Attacks: a Phase 1, Randomized, Open-Label, Fixed-Sequence, 2-Period Study"	39	48 (23-71)	F:28 M:11	NA	Zavegepant exposure during a migraine attack vs between attacks following a 10 mg dose.	Exposure was reduced by 17% for C_max _and 10% for AUC0-inf during a migraine attack.
Bertz et al., 2022 [15] “Effects of Zavegepant and Concomitant Sumatriptan on Blood Pressure and Pharmacokinetics in Healthy Adult Participants”	42 dosed: zavegepant (36) Placebo (6)	31 (21-49)	F: 18, M: 24	NA	Effect of coadministration of sumatriptan and zavegepant on blood pressure and pharmacokinetic properties	Coadministration of sumatriptan and Zavegepant did not show any significant differences in blood pressure or PK
Lipton et al., 2023 [16] “Safety, Tolerability, and Efficacy of Zavegepant 10 mg Nasal Spray for the Acute Treatment of Migraine in the USA: a Phase 3, Double-Blind, Randomized, Placebo-Controlled Multicentre Trial”	1978 enrolled, 1405 randomized; Treated Participants: Zavegepant 10 mg: 629, placebo: 653	40.8 (13.32)	F: 1059, M: 223	Yes: 171, No: 1111	Pain freedom and freedom from most bothersome symptom two hours post-dose	Zavegepant showed greater efficacy as compared to placebo in treating migraine according to the measured endpoints
Croop et al., 2022 [17] “Zavegepant Nasal Spray for the Acute Treatment of Migraine: A Phase 2/3 Double-Blind, Randomized, Placebo-Controlled, Dose-Ranging Trial”	2154 enrolled, 1673 randomized (1:1:1:1) into 4 groups: 5 mg, 10 mg, 20 mg zavegepant, or placebo	40.8 (12.66)	F: 1354 M: 234	Yes: 216, No: 1372	Pain freedom and freedom from most bothersome symptom two hours post-dose	Zavegepant showed greater efficacy as compared to placebo in treating migraine according to the measured endpoints
Biohaven Pharmaceuticals, 2023 [18] “Long-term Safety Study of BHV-3500 (Zavegepant) for the Acute Treatment of Migraine”	974 enrolled, 900 in the observation-al phase, 603 received zavegepant	42.1 (12.46)	F: 517 M: 86	NA	Adverse events, severe adverse events, and the number of participants with significant lab abnormalities	7/603 participants had severe adverse events

**Figure 2:**
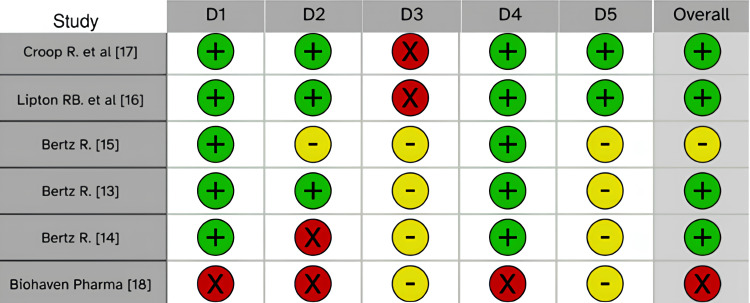
Risk of bias assessment D1: selection bias; D2: performance bias; D3: detection bias; D4: attrition bias; D5: reporting bias X (red) = High, - (yellow) = Some Concerns, + (green) = Low

The quality of the studies [13-17] was evaluated using an online risk of bias assessment tool (Figure 2).

Outcomes

The primary outcomes of efficacy were freedom from pain and freedom from Most Bothersome Symptoms (MBS) two hours post-dose. The outcomes of safety were the number of adverse events and significant adverse events. One study evaluated the safety of zavegepant by comparing the effect of coadministration of sumatriptan and zavegepant versus sumatriptan alone on the blood pressure of patients. Pharmacokinetics were measured using maximum concentration (C_max_), area under the curve (AUC), and the minimum dose required to produce CGRP inhibition.

Results

Pharmacokinetics

Bertz et al. conducted two phase 1, single-center, placebo-controlled trials in healthy fasted adults to determine the safety, tolerability, and pharmacokinetics of single and multiple ascending doses of zavegepant. This study reported no adverse effects. C_max_ and AUC were found to be proportional to the dose ranging from 5-40 mg. Single doses ≥ 10 mg produced CGRP inhibition ≥ 90%, which may be efficacious in adults. The study reported no clinically significant changes in heart rate and ECG parameters following the use of zavegepant [13].

A multicenter, single-dose, fixed-sequence, comparative bioavailability study conducted by Bertz et al assessed the correlation between zavegepant exposure and therapeutic response. Participants were randomized into two groups to receive either 10 mg or 20 mg of intranasal zavegepant. This study reported a decrease in the mean plasma concentration of zavegepant during migraine attacks as compared to concentrations between attacks. A decrease of 17% for C_max_ and 10% for AUC0-inf was observed during migraine attacks as compared to between attacks for the 10 mg dose, and a decrease of 28% and 27% was observed for C_max_ and AUC0-inf, respectively, for the 20 mg dose. The study reported no exposure-related differences in efficacy endpoints, i.e., pain-free, pain relief, and functional disability scores [14].

Bertz et al. conducted a phase 1, single-center, placebo-controlled trial that assessed the safety and tolerability of sumatriptan and zavegepant alone and in combination. The difference between the time-weighted average of mean arterial pressure for the “sumatriptan + zavegepant” group and the “sumatriptan” group was found to be 0.04 mmHg. The study reported that there were no significant changes in blood pressure or pharmacokinetics following the co-administration of sumatriptan and zavegepant as compared to sumatriptan alone [15].

Efficacy

A phase 3, double-blind, placebo-controlled, clinical trial by Lipton et al. reported that intranasal zavegepant 10 mg was efficacious in treating acute migraine and had a decent safety profile. The trial enrolled 1978 participants, of which 1405 were eligible for the study; 702 of these participants were enrolled in the placebo group and 703 in the intervention group. In the efficacy analysis, we included 1269 participants. Of these, 623 were in the 10 mg zavegepant group, and 646 in the placebo group. The primary endpoints for efficacy were taken as pain freedom two hours post-dose and freedom from MBS two hours post dose. Of participants in the 10 mg zavegepant group and in the placebo group, 23.6% and 14.9% had freedom from pain two hours post dose, respectively. Furthermore, 39.6% of participants in 10 mg zavegepant and 31.1% of placebo participants had freedom from MBS two hours post dose. Secondary outcome measures for efficacy including pain relief, photophobia freedom, phonophobia freedom, and return to normal functionality showed that 10 mg of zavegepant was efficacious in treating migraine [16].

Croop et al. conducted a randomized, placebo-controlled, dose-ranging, phase 2/3 clinical trial to determine the safety and efficacy of different doses of zavegepant. This trial reported similar outcome measures for efficacy as Lipton et al. A total of 2154 participants were enrolled for this study out of which 1673 were randomized (1:1:1:1) into four groups, i.e., zavegepant 5 mg, 10 mg, 20 mg, and placebo. As for the primary outcome measures of efficacy, 19.6% of the participants in the 5 mg group, 22.5% in 10 mg, 23.1% in 20 mg, and 15.5% in the placebo group reported freedom from pain two hours post dose. Furthermore, 39% of participants in the 5 mg, 41.9% in the 10 mg, 42.5% in the 20 mg, and 33.7% in the placebo group reported freedom from MBS at two hours post dose. Additionally, secondary outcome measures for efficacy including pain relief, photophobia freedom, phonophobia freedom, and return to normal functionality also showed that all administered doses of zavegepant were efficacious in treating migraine [17].

As for the pooled results of these two randomized control trials, there were 1014 participants in zavegepant 10 mg group and 1047 in the placebo group. The freedom from pain two hours post dose (OR 1.70, 95%CI 1.36-2.12, p<0.0001) and MBS two hours post dose (OR 1.44, 95%CI 1.20-1.73, p <0.0001) was significantly more in zavegepant 10 mg group as compared to placebo.

Safety

Croop et al. reported no serious adverse events in the 5 mg, 20 mg, or placebo groups. However, one event of thrombosis was reported in the 10 mg group out of 394 participants. Dysgeusia was reported by 13.92% of participants in 5 mg, 13.45% in 10 mg, 16.13% in 20 mg zavegepant groups, and 3.47% in the placebo group. Furthermore, 1.29% of participants in the 5 mg, 1.27% in 10 mg, 5.21% in 20 mg, and 0.25% in the placebo groups reported nasal discomfort following the administration of the respective regimen [17]. Lipton et al. reported no serious adverse events. Dysgeusia was reported in 20.51% of participants of the 10 mg zavegepant group and 4.75% of the placebo group. Both trials reported no mortalities [16].

A trial by Biohaven Pharmaceuticals to determine the long-term safety of zavegepant for the acute treatment of migraine enrolled 974 participants, of which 900 entered the observational phase and 608 entered the long-term treatment phase. Six hundred and three participants eventually received treatment with zavegepant 10 mg. A total of seven participants reported severe adverse events including bile duct stenosis, appendicitis, herpes zoster meningoencephalitis, pneumonia, concussion, fall, back pain, multiple sclerosis, and pleurisy. Other non-serious adverse events included nausea (6.14%), coronavirus disease 2019 (COVID-19) (7.46%), back pain (5.47%), dysgeusia (39.14%), nasal discomfort (10.28%), nasal congestion (5.47%), and throat irritation (5.47%) [18].

A study by Bertz et al. reported that single and multiple ascending doses of zavegepant up to 40 mg for 8-14 days were safe and well tolerated. No clinically significant effects of zavegepant on ECG patterns, morphologies, and heart rate were reported [13]. Another study by Bertz et al. showed that the coadministration of sumatriptan and zavegepant did not show any clinically significant differences in blood pressure and pharmacokinetic properties as compared to the sole administration of sumatriptan [15].

Discussion

Treatment with intranasally administered zavegepant at 10 mg dose resulted in significantly more efficient freedom from pain two hours post dose and from the MBS two hours post dose. Croop et al. also assessed the efficacy of zavegepant at 5 mg and 20 mg doses [17]. The results for 5 mg were not significant whereas those of 20 mg were. Secondary endpoints were chosen as freedom from photophobia and phonophobia two hours post dose, and relief from pain 30 minutes, 60 minutes, and two hours post dose. All the results were significant for the 10 mg dose and for 20 mg as well in the trial of Croop et al. [17]. All these factors support zavegepant nasal spray as an effective acute treatment for migraine.

One of the major aims of migraine treatment is reducing disease-related disability. Migraine is the second most common cause of disability in the world concerning years lived with disability and is the top one among young women [19]. Both Lipton et al. and Croop et al. reported a greater proportion of participants in the zavegepant group returning to normal function at 30 minutes, 60 minutes, and two hours post dose. Return to normal function was measured as the proportion of participants reporting normal function in the functional disability (mildly impaired, severely impaired, or requires bedrest) subgroup at the time of dosing.

Patients with chronic migraine experience a wide variety of symptoms, such as emotional distress, non-cephalic pain, sleep problems, and gastrointestinal upset [20]. CGRP-targeted monoclonal antibodies have been shown to have a favorable outcome on the management of chronic migraine [21]. The efficacy, safety, and tolerability of atogepant in preventing chronic migraine were investigated in a phase 3, multicenter, randomized, double-blind trial, in which the primary goal of migraine treatment was a reduction in the mean number of monthly migraine days [22], which aims to increase the sustained relief and freedom from pain. In both of the included trials, the zavegepant group showed significantly increased relief and freedom from pain at 2-24 hours, and 2-48 hours post dose [16,17]. These findings further corroborate the efficacy of zavegepant in the treatment of migraine.

Zavegepant is administered as an intranasal spray. There have been many other intranasal drugs used for migraine before such as dihydroergotamine mesylate [23], sumatriptan [24], and butorphanol [25]. Intranasal administration has some advantages over other delivery methods. For example, it bypasses the small intestine's absorption process, which can be delayed during a migraine attack's acute phase. Patients who experience nausea may prefer non-oral formulations as they decrease the chance of vomiting and are more rapidly effective [26]. There is also some evidence that the oral form of triptans causes more nausea than nasal sprays. The zavegepant nasal spray may prove to be a superior form of treatment to oral atogepant [27]. Moreover, intranasal sprays have a shorter T_max_ than their respective oral forms, which results in a rapid onset of action [28]. Zavegepant nasal spray has a T_max_ of approximately 30 minutes for doses ranging from 5 mg to 40 mg.

The intranasal spray of zavegepant is usually well tolerated and shows a favorable safety profile at doses of 5 mg, 10 mg, and 20 mg. One study increased the dose up to 40 mg, but it was well tolerated as well [13]. The most common adverse events include dysgeusia, nasal discomfort, and nausea and the majority of adverse events were either mild or moderate [13,16,17]. Moreover, zavegepant nasal spray did not increase levels of aminotransferases or total bilirubin more than three times the normal range or show any other signs of hepatoxicity [13]. In the trial by Croop et al., participants did show five significant adverse events, but none of them was considered related to treatment [17]. The effects of zavegepant on ECG were assessed in a trial and its results concluded that zavegepant had no clinically significant effects on the parameters of ECG [13]. Another trial assessed the effects of zavegepant co-administered with sumatriptan versus sumatriptan alone on blood pressure and found no significant difference. They deemed both treatments to have a favorable safety profile and be well tolerated [15]. The side effects of triptans include dry mouth, a feeling of heaviness, flushing, dizziness, nausea, and if you take a shot of triptan, a skin reaction. These are usually mild adverse effects and go away without any additionally needed treatment [29]. Available data suggest that the efficacy of triptans is essentially similar to those of second-generation gepants and that the gepants are more tolerable [30]. Future studies are needed, however, to assess their safety in long-term usage.

Newly developed drugs that act on the CGRP pathway herald the dawn of a new era in the management of migraine. CGRP is a 37 amino acid neuropeptide that plays a significant role in the pathophysiology of migraine. The blockade of CGRP alleviates neural inflammation and it has also been reported to reduce pain sensation. The effect can be blocked by either targeting the CGRP ligands or the CGRP receptors. Two types of modalities have been designed: the small molecules (gepants), and the monoclonal antibodies (MAbs) [31]. Ubrogepant and Rimegepant are second-generation gepants and have shown efficacy as abortive treatments [32]. Atogepant is the only oral and also the first CGRP antagonist developed to prevent migraine attacks [33]. Zavegepant is another third-generation drug and is the first intranasal CGRP antagonist to be developed. It can also be administered via oral and subcutaneous routes; however, the intranasal route provides the most rapid onset of action [34]. These gepants have also been shown to be better than CGRP or CGRP receptor-directed monoclonal antibodies, which are also used for migraine management [33]. The monoclonal antibodies are large molecules, and they have to be administered via IV or subcutaneous injections. Furthermore, they have long half-lives and thus they’re administered once a month. Compared to them, the small molecules are administered orally and have shorter absorption and elimination phases. Thus, they have to be administered daily [35].

## Conclusions

Our systematic search of three databases yielded a total of six studies that fit our inclusion criteria. Forest plots were not reported as only two out of these six studies reported data that was poolable. The phase 2/3 and phase 3 clinical trials included in our systematic review both reported that intranasal zavegepant was efficacious in the treatment of migraine. Both trials did, however, report dysgeusia as an adverse event in some participants. The remaining studies included in our review also reported that zavegepant was beneficial in the treatment of migraine and that it does not cause any significant differences in ECG patterns or mean arterial pressure values when compared with sumatriptan. Overall, zavegepant is a promising new candidate for the treatment of migraine headaches, especially for nauseated patients or those who have difficulty swallowing. An intranasal spray for migraine treatment can also improve the overall quality of life of patients living with this condition by reducing the need for taking regular pills or tablets. However, the majority of included studies compared zavegepant efficacy with a placebo; hence, there is a requirement for clinical trials to determine the comparative safety and efficacy of zavegepant and other medications for migraine headaches. 
